# The coexistence of terms to describe the presence of multiple concurrent diseases

**DOI:** 10.15256/joc.2013.3.22

**Published:** 2013-10-08

**Authors:** José Almirall, Martin Fortin

**Affiliations:** ^1^Department of Family Medicine and Emergency Medicine, Université de Sherbrooke, Sherbrooke, Québec, Canada, and Centre de santé et de services sociaux de Chicoutimi, Chicoutimi, Québec, Canada

**Keywords:** Comorbidity, definitions, multimorbidity, multiple conditions, terminology

## Abstract

**Background:**

Consensus on terminology for multiple diseases is lacking. Because of the clinical relevance and social impact of multiple concurrent diseases, it is important that concepts are clear.

**Objective:**

To highlight the diversity of terms in the literature referring to the presence of multiple concurrent diseases/conditions and make recommendations.

**Design:**

A bibliometric analysis of English-language publications indexed in the MEDLINE database from 1970 to 2012 for the terms *comorbidity, multimorbidity, polymorbidity, polypathology, pluripathology, multipathology,* and *multicondition*, and a review of definitions of *multimorbidity* found in English-language publications indexed from 1970 to 2012 in the MEDLINE and SCOPUS databases.

**Results:**

*Comorbidity* was used in 67,557 publications, *multimorbidity* in 434, and the other terms in three to 31 publications. At least 144 publications used the term *comorbidity* without referring to an index disease. Thirteen general definitions of *multimorbidity* were identified, but only two were frequently used (91% of publications). The most frequently used definition (48% of publications) was “*more than one or multiple chronic or long-term diseases/conditions*”. Multimorbidity was not defined in 51% of the publications using the term.

**Conclusions:**

*Comorbidity* was overwhelmingly used to describe any clinical entity coexisting with an index disease under study. *Multimorbidity* was the term most frequently used when no index disease was designated. Several definitions of *multimorbidity* were found. However, most authors using the term did not define it. The use of clearly defined terms in the literature is recommended until a general consensus on the terminology of multiple coexistent diseases is reached.

Journal of Comorbidity 2013;3:4–9

## Introduction

“*Although patients with more than one diagnosed disease are frequently encountered in modern medical practice, the inter-relationships and effects of multiple diseases have not received suitable taxonomic attention in clinical science*”. This is how Alvan R. Feinstein begins his seminal paper, published in 1970, in which he coined the term *comorbidity* as “*any distinct additional clinical entity that has existed or that may occur during the clinical course of a patient who has the index disease under study*” [1], understanding, by disease, a definite pathologic process with a characteristic set of signs and symptoms. Although written more than 40 years ago, this opening line is still quite actual and valid. The terms and definitions used in the context of multiple concurrent diseases have not received suitable attention in the literature to date. For example, the term *comorbidity,* which was well defined by Feinstein, has been used (and sometimes redefined) to describe the coexistence of multiple diseases without considering an index disease. Other definitions of *comorbidity* found in the literature that do not refer to an index disease are: *“the association of two distinct diseases in the same individual at a rate higher than expected by chance*” [2]; “*three or more clinical conditions*” [3]; “*the presence of more than one clinical condition*” [4]; “*the co-existence of multiple chronic conditions in a single individual*” [5]; “*more than one chronic disease*” [6]; “*several chronic conditions simultaneously*” [7]; “*the concurrence of multiple health conditions in the same person*” [8]; “*multiple chronic conditions*” [9]; “*two or more diseases, with distinct aetiopathogenesis (or, if the aetiology is unknown, with distinct pathophysiology of the organ or system), that are present in the same individual in a defined period of time*” [10].

Inconsistency and ambiguity in the use of the terms and implications for practice and research were pointed out by van den Akker and colleagues in 1996 [11]. They proposed to use the term *multimorbidity* in situations where no index disease is considered, and to reserve *comorbidity* for situations in which one of the diseases is considered as index, as originally defined by Feinstein [1, 11]. Despite van den Akker and colleagues’ call for a conceptual organization, and also more recent efforts in this sense [12, 13], ambiguity still persists. There is no general agreement among authors; various terms can be found in the medical literature: *comorbidity*, *multimorbidity, polymorbidity, polypathology, pluripathology, multipathology, multicondition*.

All terms intuitively refer to the presence of multiple diseases, but the exact meaning or definition, if and when provided, varies from one author to another. In addition to a lack of general agreement in the definition of terms, it is not uncommon to see them used without any definition, which may imply that many authors presume that the meaning of the term is commonly understood.

This diversity may have a negative impact on practice and research. Because of the different choices made in the design of studies with regard to terms (see list above), to diseases (index disease or not; only chronic or also acute; distinct or related pathophysiology), the number of diseases considered as *multiple* (two, three, four, five), the use of indices (definitions based on scores), it may be difficult, on the one hand, to find the information of interest, and to compare the results on the other. A lack of a universal consensus on terminology and definitions is an obstacle to the dissemination of information and the application of new knowledge. Because of the clinical relevance and social impact of the presence of multiple diseases, it is important that the conceptualization of this subject is clear and without ambiguities. Thus we conducted a bibliometric analysis of terms used in the context of multiple diseases and a review of definitions of the term *multimorbidity*. The objective of this review was to shed light on the diversity of terms and the frequency of their use, to identify the different definitions of *multimorbidity*, and to provide recommendations for the use of terms.

## Methods

For the bibliometric analysis of terms used when referring to the presence of multiple conditions in one individual, we searched the literature using the MEDLINE database in PubMed (http://www.ncbi.nlm.nih.gov/pubmed/). We chose this database because it contains more than 22 million citations for biomedical literature, is freely accessible, and is widely used in bibliographical searches. We used the following search terms: *comorbidity* (and its variant *co-morbidity*)*, multimorbidity* (and its variant *multi-morbidity*)*, polymorbidity* (and its variants *poly-morbidity* and *polymorbidities*)*, polypathology* (and its variants *poly-pathology* and *polypathologies*)*, pluripathology, multipathology* (and its variants *multi-pathology* and *multipathologies*), and *multicondition*. This list was generated based on our personal experience of reading publications on multiple concurrent diseases for several years. Other terms that we are not aware of may exist. We searched for the terms in all fields of the publications (title, abstract, etc.); the only filters used were: (1) publications written in English, and (2) publications indexed from 1970 to December 2012. In the case of *comorbidity*, the number of publications indexed under that term was overwhelming. To reduce the number of publications in our list and to target literature in which the subject heading is considered the main idea of the article, the filter “MeSH Major Topic” was additionally used for the term *comorbidity*. All types of publication (research articles, editorials, opinion papers, etc.) were included in our review. No further steps were taken to find publications that the automatic research engine could have missed. We counted the number of publications in which each term was used. In publications using a term for the first time, we also took note of the reference and country of origin of the work as we thought that there could be a link between the mother tongue of the authors and the introduction of new terms used to describe the presence of multiple coexistent conditions.

To conduct a review of definitions for the term *multimorbidity* (and its variant *multi-morbidity*), we used the MEDLINE database in PubMed and the SCOPUS database accessed through the library of the Université de Sherbrooke. In the MEDLINE database, we searched for the terms in all fields of the publications; the only filters used were: (1) publications written in English, and (2) publications indexed from 1970 to December 2012. In the SCOPUS database, we used the same filters, but also considered all subject areas contained in the database: life sciences, health sciences, physical sciences, and social sciences and humanities. All types of publication (research articles, editorials, opinion papers, etc.) were included in our review. For the purpose of this review, we looked for the digital or hard copy of the publications identified. We read all available publications looking for a definition or a description of the term. We extracted all definitions provided.

## Results

In the case of *comorbidity*, the bibliographic search initially yielded a total of 67,557 publications. After applying the filter “MeSH Major Topic”, 1,028 publications remained. We read the abstracts to assess how many of these publications using the term *comorbidity* were papers in which an index disease or medical condition was under study. A total of 162 publications did not have an abstract. In the remaining 866 publications with abstracts, 144 (17%) did not have an index disease or medical condition under study, suggesting that the term *comorbidity* was not used as originally described by Feinstein.

**Figure 1:**
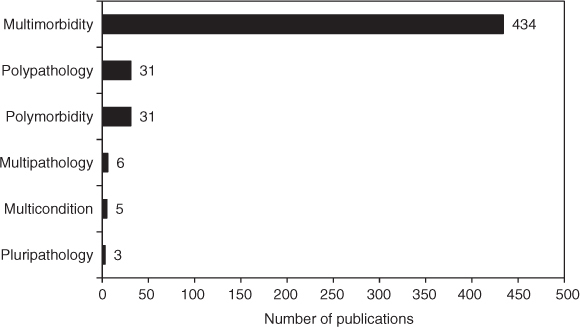
Terms other than *comorbidity* used in the context of multiple diseases or conditions and number of publications using them in the medical literature in English. The term *comorbidity* was not included in the figure due to the disproportionally high number of publications using the term (see text).

Results of the bibliometric analysis of all other terms (excluding *comorbidity*) are shown in Figure 1. Of the terms shown in Figure 1, the one most often used was *multimorbidity* (434 publications). The other terms were used in three to 31 publications. The reference and country of origin of publications using each term for the first time are shown in Table 1 [14–20]. Only the terms *multipathology* and *multicondition* were used for the first time in publications from an English-speaking country.

In the review of definitions of *multimorbidity* using the MEDLINE and SCOPUS databases, we identified 465 publications that used the term. It was not possible to obtain the digital or hard copy of four publications. We carefully read the remaining 461 publications looking for definitions. There were 233 publications (51%) in which the term *multimorbidity* was used without providing any definition of the term. In the publications providing a definition, we did not consider the exact wording to describe *multimorbidity* as a unique definition of the term. Instead, we grouped definitions with similar meanings into categories of general definitions. We identified 13 general definitions of *multimorbidity*, but two of them were predominantly used. Table 2 summarizes the different definitions of *multimorbidity* used in the literature, as well as the number of publications that used them. The most frequently used definition of *multimorbidity* was “*more than one or multiple chronic or long-term diseases/conditions*” (including either *physical or mental diseases, or both*, in the definition), which was closely followed by “*more than one disease or condition*” without specifying *chronic or long-term* in the definition.

## Discussion

The bibliometric analysis of terms referring to the presence of multiple diseases in one individual demonstrates the diversity of terms used in the medical literature to describe this common situation, and differences in the frequency of their use. This diversity of terms is not appropriate for medical language in which unambiguous well-defined terms are needed. Apart from *comorbidity*, only two terms (*multipathology* and *multicondition*) were used for the first time in publications from an English-speaking country. This raises the question whether this diversity of terms has originated from the translation of studies originally conducted in other languages into English.

As expected, the term *comorbidity* was the most often used when referring to the presence of multiple diseases in one individual. However, we determined that 17% of the revised abstracts were papers without an index disease under study, suggesting that the use of the term differed from Feinstein’s original definition [1]. The second most frequently used term was *multimorbidity*. We found that this term was most often used in the context of multiple coexistent diseases or conditions without designating an index disease. This prompted us to conduct a more detailed review of the literature using the term *multimorbidity* and the definitions proposed by different authors. As shown in Table 2, we identified 13 general definitions of *multimorbidity*, but the vast majority of publications used only two of them. An important number of publications using the term (51%) did not provide any definition. The authors of these publications may have presumed that the meaning of the term was generally understood.

Finding different definitions for *multimorbidity* led us to another conceptual situation: the use of the terms *disease*, *condition,* or *illness* to describe the health issues of patients. The term *illness* is frequently used as a synonym for disease, but in many cases it refers to the patients’ personal experience of their disease. As mentioned previously, the term *disease* refers to a defined pathologic process with a characteristic set of signs and symptoms. *Condition* is a broad term that includes *disease*, but also other health issues that fall outside of the traditional disease model. The terms *disease* and *condition* were overwhelmingly used in the definitions of *multimorbidity* and seem to be more appropriate for describing the coexistence of health issues.

### What to do with so many terms?

For the time being, since there is no generally accepted terminology, we should be aware of the coexistence of the many terms, considering that definitions for any given term may vary. A comprehensive search of information on the subject should include all terms. If one is writing an article, our recommendation is that only one term be used to avoid ambiguity and to clearly define the meaning of the term used.

General agreement about terminology and definitions should be reached. We suggest that only two terms be used, one for situations in which multiple diseases/conditions coexist with an index disease under study, and another for situations in which there are multiple coexistent diseases/conditions, but none considered as the index disease/condition. In the first case, the term *comorbidity* already exists and is widely accepted and used. Indeed, at the time of writing this text, it is the only term, of those mentioned above, that is accepted as a Medical Subject Heading (MeSH) (Tree numbers: N05.715.350.225; N06.850.490.687) by the US National Library of Medicine (http://www.nlm.nih.gov/mesh/MBrowser.html), and is defined as “*the presence of co-existing or additional diseases with reference to an initial diagnosis or with reference to the index condition that is the subject of study…*”. Redefining or using the term *comorbidity* for other purposes, particularly in situations where no index disease is considered, should be avoided. Publications in which *comorbidity* is used with a different meaning from the one defined by Feinstein [1] and adopted by the US National Library of Medicine would be very difficult to find among thousands of other articles using the term as originally defined. The information contained in publications in which *comorbidity* was redefined to fit other situations may not reach the intended audience.

With regard to the situation in which there are coexistent diseases, but none considered as an index disease, we recommend using the term that is most widely used, that is, *multimorbidity*.

### Which definition of multimorbidity to use?

The lack of consensus about a definition of *multimorbidity* implies that authors may use any definition. However, it should be noted that the high prevalence of multiple diseases/condition these days is generally considered as the result of aging of the population and the growing proportion of patients with coexistent long-term diseases/conditions, although it is clear that the presence of multiple chronic diseases/conditions is not a problem only affecting older patients [21, 22]. Indeed, all epidemiologic and clinical studies using the term *multimorbidity* that we reviewed were about long-term diseases or conditions. Therefore, the most frequently used general definition of “*multiple co-occurring chronic or long-term diseases or conditions, including both physical and mental diseases, and none considered as index disease”*, seems a reasonable choice to define *multimorbidity*.

### Possible future actions

We believe that a general consensus on the terminology for multiple coexistent diseases should exist, as well as a general definition of *multimorbidity*, although we acknowledge that there are authors who might have a different opinion on this. Addressing our words to those sharing our belief in this matter, we think that a first step towards a consensus is to expose the situation and instigate reflection on this, which is one of the intentions of this work. This could be followed by an exchange of ideas including as many people as possible interested in the subject, to end with a consensus that could be followed by a proposal of a MeSH. With regard to a joint definition of multimorbidity, we are aware of the recent effort of a large European research team that undertook a methodic data extraction and proposed a definition that was not included in our list of definitions up to 2012 because it was published in 2013 [13]. The proposed definition of *multimorbidity* was: “*any combination of chronic disease with at least one other disease (acute or chronic) or biopsychosocial factor (associated or not) or somatic risk factor*”. However, the definition lacks the input from many well-known experts from all over the world, including Europe, who have authored an important number of publications on the subject and whose opinions would have been very valuable in the development of the definition.

### Study limitations

A limitation of any bibliographic search is the potential omission of relevant articles. For the bibliometric analysis, we did not try to be exhaustive, but rather highlight the use of multiple terms, when referring to multiple diseases, as well as to provide an estimate of their frequency of use. We considered that one database, such as MEDLINE, was enough for these purposes. In the review of definitions of *multimorbidity*, we used only one additional database (SCOPUS) to retrieve publications, and this is a limitation of the study. We chose the SCOPUS database because, in addition to covering MEDLINE 100%, it includes more than 4,300 titles from the life sciences, more than 5,300 titles in social sciences and humanities, and more than 7,200 titles in physical sciences. The use of SCOPUS in addition to MEDLINE increased the number of publications identified as using the term *multimorbidity* from 434 to 465. Although we tried to be exhaustive with the two databases, it is possible that we were unaware of other relevant publications. Our search strategy was not limited to MeSH or keywords assigned by authors to avoid missing publications that were not indexed under these terms. However, as we were searching for specific terms, our search surely missed publications on the topic of multiple concurrent diseases that did not use any of the terms in our list.

## Conclusions

There are many terms currently used in the context of multiple concurrent diseases. This diversity may have a negative impact on practice and research. The term *comorbidity* should be reserved for situations in which one or more diseases coexist with an index disease under study. For situations in which there are multiple coexistent diseases, but none considered as index disease, the term *multimorbidity* was by far the most frequently used. The term *multimorbidity* was most often defined as “*the presence of more than one or multiple chronic or long-term diseases or conditions*”. We recommend to clearly define terms until a general consensus on terminology of multiple coexistent diseases is reached.

## Figures and Tables

**Table 1:** List of English terms used in the context of multiple coexistent diseases/conditions (excluding comorbidity) with reference and country of origin of publications using each term for the first time.

Term	Reference	Country of origin
Multimorbidity	Weyerer (1983) [14]	Germany
Polymorbidity	(1) Ulsperger and Hofbauer (1992) [15] (2) Pumprla *et al*. (1992) [16]	(1) Austria (2) Czech Republic
Polypathology	Touitou *et al*. (1984) [17]	France
Pluripathology	Hertzeanu and Aron (1985) [18]	Israel
Multipathology	Butler (1981) [19]	USA
Multicondition	Ahluwalia *et al*. (2005) [20]	USA

**Table 2:** General definitions of multimorbidity and number of publications using them.

Definition*	Publications (*n*)
More than one or multiple chronic or long-term diseases/conditions	109
More than one or multiple diseases or conditions (no other specification)	99
More than one or multiple chronic and/or acute diseases	7
Vascular multimorbidity (presence of clinically relevant disease in at least two major vascular territories manifest at the same time in a single patient)	4
Five or more coexisting, moderate to severe physical illnesses	1
Impairment of three or more organ systems	1
Multiple disease states leading to loss in daily functioning	1
The total burden of biological dysfunction	1
The co-occurrence of comorbidities, functional limitations, and/or geriatric syndromes	1
Neoplastic multimorbidity (more than one primary cancer in the same person)	1
The co-occurrence of states or situations related to the following health domains: medical comorbidity, musculoskeletal, general physical and social functioning	1
Presence of morbidity in two or more domains of the Cumulative Illness Rating Scale	1
Charlson Index Score >1	1

*Definitions with similar meaning were included within the same group of general definition.
